# Safety and pharmacodynamics of the ferroportin inhibitor vamifeport in patients with non-transfusion-dependent β-thalassemia: results from a randomized phase 2a study

**DOI:** 10.1186/s13023-025-04119-y

**Published:** 2025-11-25

**Authors:** Antonis Kattamis, Ali Taher, Vip Viprakasit, Carina Levin, Ricardo Hermosilla, Peter Szecsödy, Frank Richard, M. Domenica Cappellini, John Porter

**Affiliations:** 1 Thalassemia Unit, First Department of Pediatrics, National and Kapodistrian University of Athens, ‘Aghia Sofia’ Children’s Hospital, Goudi, Athens 11527 Greece; 2 Division of Hematology and Oncology, Department of Internal Medicine, American University of Beirut Medical Center, Beirut, Lebanon; 3 Siriraj Hospital, Mahidol University, Bangkok, Thailand; 4 Pediatric Hematology Unit, Emek Medical Center, Afula, 1834111 Israel; 5 The Ruth and Bruce Rappaport Faculty of Medicine, Technion–Israel Institute of Technology, Haifa, Israel; 6 CSL Behring, Berne, Switzerland; 7 CSL Vifor, Flughofstrasse 61, Glattbrugg, 8152 Switzerland; 8 Present Address: Heidelberg Pharma AG, Gregor-Mendel Strasse 22, 68526 Ladenburg, Germany; 9 Fondazione IRCCS, Ca Granda Policlinico, University of Milan, Milan, Italy; 10 University College London, London, UK

**Keywords:** Β-thalassemia, Iron, Tolerability, Transferrin saturation, Vamifeport

## Abstract

**Background:**

Non-transfusion-dependent beta-thalassemia (β-NTDT) is characterized by ineffective erythropoiesis, increased intestinal iron absorption, and iron overload. The ferroportin inhibitor, vamifeport, has been shown to improve erythropoiesis via decreases in serum iron and transferrin saturation levels in preclinical models and healthy volunteer studies.

**Objective:**

The objective of this 12-week, double-blind, randomized, placebo-controlled, phase 2a study was to assess the safety and tolerability of vamifeport versus placebo in adults with β-NTDT (primary endpoint). Iron-related pharmacodynamic effects (preliminary efficacy) were also assessed as a secondary endpoint.

**Methods:**

Randomized, adult patients weighing 40–59 kg and 60–100 kg received vamifeport 60 mg and 120 mg (once [QD] or twice [BID] daily), respectively, for 12 weeks. Non-transfusion-dependent thalassemia was defined as transfusion requirements < 5 units of red blood cells during the 24 weeks before randomization.

**Results:**

Twenty-five patients were included (vamifeport QD *n* = 9, BID *n* = 12; placebo *n* = 4); 64% were male and 56% weighed < 60 kg. Baseline serum iron and transferrin saturation levels were similar across treatment groups. All treatment-emergent adverse events were mild/moderate, and rates were similar across groups (vamifeport QD 67%, BID 58%; placebo 75%). There were no deaths or serious treatment-emergent adverse events and no clinically relevant changes in safety parameters. Serum iron and transferrin saturation levels decreased by 2 h after the first vamifeport dose (mean [standard deviation] decreased QD − 12.2 [6.5], BID − 14.5 [12.1] µmol/L and QD − 33.6 [18.9], BID − 37.2 [27.6] %, respectively) and remained below baseline levels at each subsequent visit. There were no clinically meaningful changes in the placebo group.

**Conclusion:**

In this 12-week study, vamifeport had a favorable safety/tolerability profile, with no changes in hemoglobin levels ≥ 1.0 g/dL, and promising pharmacodynamic effects versus placebo in adults with β-NTDT.

**Trial registration:**

ClinicalTrials.gov, NCT04364269. Registered 01 April 2020; Prospectively registered, https://clinicaltrials.gov/study/NCT04364269?term=NCT04364269&rank=1.

**Supplementary Information:**

The online version contains supplementary material available at 10.1186/s13023-025-04119-y.

## Introduction

Beta (β)-thalassemia is an inherited hemoglobin disorder characterized by an imbalanced α/β-globin chain ratio, ineffective erythropoiesis, and chronic hemolytic anemia [1, 2]. The imbalanced production of α/β-globin chains leads to the development of insoluble α-globin aggregates on erythroid precursors and red blood cell (RBC) membranes, the release of free, non-transferrin-bound iron (NTBI), and the formation of reactive oxygen species, resulting in premature RBC death and tissue hypoxia [2–4]. This leads to ineffective erythropoiesis, which drives compensatory hemopoietic expansion, suppressed synthesis of the iron regulatory hormone hepcidin, and increased ferroportin-mediated intestinal iron absorption and iron release from the reticuloendothelial system, which may eventually result in iron overload [1, 2, 5]. Modulation of the hepcidin-ferroportin axis may potentially limit iron overload and improve ineffective erythropoiesis in patients with β-thalassemia [5].

Patients with β-thalassemia are generally categorized according to their requirement for blood transfusions, although the underlying disease process remains similar irrespective of this. Unlike those with transfusion-dependent thalassemia, patients with non-transfusion-dependent thalassemia (NTDT) do not require regular transfusions for survival but may require occasional or even periodic transfusions in certain clinical scenarios, which can contribute to secondary iron overload and the need for iron-chelation therapy (ICT) [2, 6].

Vamifeport (VIT-2763) is a novel, oral, small-molecule ferroportin inhibitor [5] that has been shown to improve anemia, ineffective erythropoiesis, and dysregulated iron homeostasis in animal disease models [7, 8]. Vamifeport inhibits ferroportin-mediated iron export into plasma and decreases serum iron and transferrin saturation (TSAT) levels in the Hbbth3/+ mouse model of β-thalassemia [7, 8]. This drug was also shown to be well tolerated and to temporarily reduce serum iron and TSAT levels in a Phase 1 study in healthy volunteers [9]. The aim of the present phase 2a study was to assess the safety, tolerability, and pharmacodynamics of vamifeport versus placebo in patients with β-NTDT over a 12-week treatment period.

## Methods

### Study design, patients, and treatment

This phase 2a, double-blind, randomized (via a validated interactive web response system), placebo-controlled, parallel group, multicenter study (Clinicaltrials.gov identifier NCT04364269) included males and females aged 18–65 years with a documented β-NTDT (i.e., β- or hemoglobin [Hb] E/β-thalassemia) diagnosis. In this study, non-transfusion dependency was defined as receipt of <5 units of RBC during the 24 weeks before randomization. Patients were eligible to participate if they had a mean baseline hemoglobin level of ≤ 11 g/dL on at least 2 consecutive measurements ≥ 1 week apart, within the 6 weeks prior to randomization. Use of ICT within 4 weeks before randomization was not permitted; patients with TSAT < 30%, serum ferritin < 150 ng/mL, or estimated glomerular filtration rate < 30 mL/min/1.73 m2 were excluded. Patients with transfusion-dependent β-thalassemia or compound heterozygosity for sickling phenotype variants such as HbS/β-thalassemia or HbC disease, or HbH disease were excluded. Eligible subjects were centrally allocated a randomization number just prior to dosing, following the randomization schedule generated by the Biostatistics Department of Medidata/RTSM. Patients were to be recruited from approximately 20 primary care sites across 5 countries (Greece, Israel, Italy, Lebanon, and Thailand). A 2-cohort design was initially planned, with adolescent subjects aged 12–17 years to be included in a separate cohort. However, the second cohort was canceled due to recruitment issues associated with the COVID-19 pandemic and the lack of an observed benefit regarding a hemoglobin increase of > 1 g/dL in any treatment arm on a blinded review of the study data.

The study comprised a 4-week screening period, a 12-week treatment period, and a 4-week safety follow-up period. Patients were randomized in an 8:8:4 ratio using an interactive web response system to receive oral vamifeport once (QD) or twice daily (BID) or a matching placebo. Randomization to active treatment was based on the patient’s body weight. Patients with a body weight of 40–59 kg received vamifeport at a dose of 60 mg QD or 60 mg BID, or placebo; patients with a body weight of 60–100 kg received vamifeport 120 mg QD or vamifeport 120 mg BID or placebo. Study medication and/or a matching placebo was administered BID to all patients to maintain the study blinding.

### Ethics declaration

The study was conducted according to the principles of the Declaration of Helsinki [10], the International Council for Harmonisation guidelines for Good Clinical Practice [11], and all relevant local, federal, or country regulatory requirements. All participants provided written informed consent to participate in the study.

### Study endpoints

The primary safety endpoint was the incidence of treatment-emergent adverse events (TEAEs) and serious adverse events by preferred Medical Dictionary for Regulatory Activities term, severity (mild, moderate, severe), and relationship to study drug. Changes in vital signs, clinical laboratory safety tests, electrocardiogram readings, and physical examination were also assessed. Safety was assessed at baseline and Weeks 1, 2, 4, 8, and 12 of treatment.

Secondary objectives included an assessment of the efficacy of vamifeport versus placebo with respect to their pharmacodynamic effects on iron-related parameters (i.e., absolute and change from baseline in total serum iron, transferrin, ferritin, and TSAT levels). Additional exploratory iron-related parameters were absolute and change from baseline in hepcidin levels and liver iron content (LIC) assessed via magnetic resonance imaging. Exploratory hematological endpoints included assessment of absolute and change from baseline in hemoglobin levels and mean corpuscular volume (MCV). Exploratory analyses of absolute and change from baseline in hemolytic markers (unconjugated bilirubin, lactate dehydrogenase [LDH], and haptoglobin) and erythropoietic markers (e.g., fetal hemoglobin) were also performed.

Iron-related parameters and hematological/hemolytic marker levels were collected at baseline, Day 1, and Weeks 1, 2, 4, 8, and 12. Erythropoietic marker levels were assessed at baseline, Day 1, and Weeks 2, 8, and 12. All blood samples for marker analysis were taken approximately 2 h after study drug administration and were analyzed at a central laboratory.

### Statistical analysis

No formal sample size calculation was performed. Approximately 30 patients were planned to be randomized to have approximately 24 participants completing the trial (based on a 20% dropout rate). The safety set comprised all patients who had received at least 1 dose of study medication; the full analysis set comprised all patients who received at least 1 dose of randomized treatment and had at least 1 post-baseline pharmacodynamic assessment. Patients who received transfusions during the study were censored in the efficacy analyses from the date of their first transfusion. No inferential statistics were performed comparing treatment groups; summary statistics are provided. All analyses were performed using SAS Version 9.4 or later (SAS Institute Inc., SAS/STAT, Cary, NC, USA).

## Results

### Patients

**Fig. 1:**
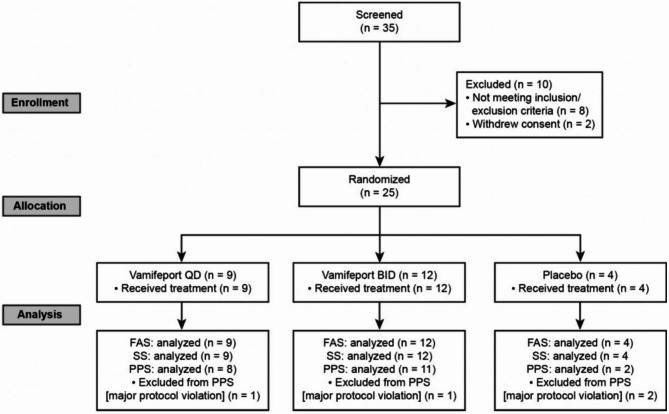
CONSORT diagram. BID, twice daily; FAS, full analysis set; QD, once daily; PPS, per-protocol set; SS, safety set

A total of 25 patients with β-NTDT were included in the study between June 11, 2020 (first patient visit), and November 3, 2021 (last patient visit), and all were included in the full analysis and safety sets (vamifeport QD *n* = 9, vamifeport BID *n* = 12, placebo *n* = 4) (Fig. 1). Two patients discontinued the study/treatment (1 patient in the vamifeport QD group [TEAE] and 1 patient in the vamifeport BID group [consent withdrawal]). There were 4 major protocol deviations: 1 patient in each of the vamifeport QD and placebo groups failed to meet inclusion criteria because they did not have a diagnosis of NTDT; and 1 patient in each of the vamifeport BID and placebo groups were deemed as non-compliant with study drug (patient adherence less than 80%). Overall, 9 patients were included from sites in Thailand, 7 from Greece, and 3 patients each from Lebanon, Israel, and Italy.

Median age was 42.0 (26–61), 31.0 (18–40), and 40.5 (range 34–58) years in the vamifeport QD, vamifeport BID, and placebo groups, respectively (Table 1). All patients were Asian or White. Overall, 64% (16/25) were male, 56% (14/25) had a body weight < 60 kg, and 16% (4/25) had received prior ICT. The vamifeport QD group was mainly comprised of females (67%, 6/9) and the vamifeport BID group was mainly comprised of males (83%, 10/12). The imbalance in sex could have influenced the weight-based dosing; more patients in the vamifeport QD group received vamifeport 60 mg, and more patients in the vamifeport BID group received vamifeport 120 mg.

**Table 1:** Patient characteristics and iron-, hematological-, and hemolytic-related parameters at baseline (screening)

	Vamifeport QD (*n* = 9)	Vamifeport BID (*n* = 12)	Placebo (*n* = 4)
Age – median (range), years	42.0 (26–61)	31.0 (18–40)	40.5 (34–58)
Males, n (%)	3 (33.3)	10 (83.3)	3 (75.0)
Race, n (%)			
Asian	5 (55.6)	3 (25.0)	1 (25.0)
White	4 (44.4)	9 (75.0)	3 (75.0)
Body weight – mean (SD), kg	54.7 (8.5)	64.4 (13.0)	56.1 (4.5)
< 60 kg, n (%)	6 (66.7)	5 (41.7)	3 (75.0)
Prior ICT, n (%)	2 (22.2)	1 (8.3)	1 (25.0)
Prior blood transfusion, n (%)	3 (33.3)	5 (41.7)	4 (100)
Prior splenectomy, n (%)	1 (11.1)	3 (25.0)	3 (75.0)
Ongoing HU treatment, n (%)	2 (22.2)	2 (16.7)	2 (50.0)
Total serum iron – mean (SD), µmol/L	23.8 (13.4)	28.4 (9.8)	30.9 (13.3)
Serum TSAT – mean, %	69.3 (31.2)	79.0 (24.1)	83.3 (33.5)
Serum ferritin – mean (SD), µg/L	426.9 (239.6)	1133.2 (2119.1)	440.0 (321.5)
Hepcidin – mean (SD), nmol/L^a^	5.7 (4.1)	5.8 (6.4)	2.3 (3.1)
Serum Hb – mean (SD), g/L^a^	88.2 (17.5)	88.5 (14.7)	92.3 (9.8)
MCV – mean (SD), fL^a^	77.3 (15.7)	69.1 (9.8)	75.3 (10.6)
LDH – mean (SD), U/L^a^	244.3 (89.8)	332.7 (165.0)	230.3 (88.9)
Unconjugated bilirubin – mean (SD), µmol/L^a^	29.9 (18.5)	61.9 (33.5)	30.8 (28.2)
LIC^b^ – mean (SD), mg/g dry weight	14.4 (15.3)	6.8 (7.9)	7.0 (6.3)

^a^Baseline samples taken on Day 1, before administration of first dose; ^b^Data available for *n* = 2, *n* = 3, and *n* = 2 in the vamifeport QD, vamifeport BID, and placebo groups, respectively

BID, twice daily; Hb, hemoglobin; HU, hydroxyurea; ICT, iron-chelation therapy; LDH, lactate dehydrogenase; MCV, mean corpuscular volume; LIC, liver iron content; QD, once daily; SD, standard deviation; TSAT, transferrin saturation

Prior ICT was received by similar proportions of patients in the vamifeport QD (22%, 2/9) and placebo (25%, 1/4) groups; 8% (1/12) of those in the vamifeport BID group had received prior ICT. Prior blood transfusion had been received by all 4 patients (100%) receiving placebo, compared with 33% (3/9) of those receiving vamifeport QD and 42% (5/12) of those receiving vamifeport BID. Partial or total splenectomy had been performed at baseline in most patients receiving placebo (75% [3/4]) compared with 11% (1/9) and 25% (3/12) of those receiving vamifeport QD and BID, respectively. At baseline, 50% (2/4) of the patients in the placebo group were receiving hydroxyurea treatment compared with 22% (2/9) of those receiving vamifeport QD and 17% (2/12) of those receiving vamifeport BID.

Mean baseline serum iron concentrations and TSAT levels were broadly similar across treatment groups. Mean baseline serum ferritin concentrations were higher in the vamifeport BID group than in the remaining treatment groups, mainly due to a single outlier with very high serum ferritin levels (6754.0 µg/L); removal of this patient from the analysis did not meaningfully alter the study results. Median serum ferritin levels in the vamifeport QD, vamifeport BID, and placebo groups were 374.8, 442.1, and 257.4 µg/L, respectively. Baseline LIC data were only available for 2, 3, and 2 patients in the vamifeport QD, vamifeport BID, and placebo groups, respectively. The higher baseline LIC observed in the vamifeport QD group was based on 2 patients with an LIC of 3.5 mg/g and 25.2 mg/g, respectively (the latter patient also had a baseline ferritin level of 941.8 µg/L).

### Safety and tolerability – primary endpoint

The incidence of TEAEs was similar across treatment groups (vamifeport QD 67% [6/9], vamifeport BID 58% [7/12], placebo 75% [3/4]) (Table 2). TEAEs that were considered related to study treatment were reported in 22% (2/9), 33% (4/12), and 50% (2/4) of patients in the vamifeport QD, vamifeport BID, and placebo groups, respectively. All TEAEs were mild or moderate in intensity, and there were no deaths or serious TEAEs. One patient in the vamifeport QD group had a TEAE (acute hemolytic event) that was considered related to treatment and led to drug discontinuation/withdrawal from the study. Each treatment-related TEAE was reported no more than once per treatment group (Table 3). Overall, the most common type of TEAE by system organ class was gastrointestinal disorders (reported by 33% [4/12] of patients in the vamifeport BID group; 17% [2/12] of those in this group had a gastrointestinal event that was considered treatment related). There were no trends or clinically relevant changes from baseline in any assessed safety parameter, including laboratory values, vital signs, electrocardiogram readings, and physical examinations. Overall, vamifeport had a favorable safety and tolerability profile in adults with β-NTDT over the 12-week treatment period.

**Table 2:** Summary of treatment-emergent adverse events (all)

Number of patients (%)	Vamifeport QD (*n* = 9)	Vamifeport BID (*n* = 12)	Placebo (*n* = 4)
Any TEAE	6 (66.7)	7 (58.3)	3 (75.0)
Any severe TEAE	0 (0)	0 (0)	0 (0)
Any treatment related TEAE	2 (22.2)	4 (33.3)	2 (50.0)
Any TEAE leading to discontinuation	1 (11.1)	0 (0)	0 (0)
Any serious TEAE	0 (0)	0 (0)	0 (0)
Any TEAE leading to death	0 (0)	0 (0)	0 (0)

BID, twice daily; QD, once daily; TEAE, treatment-emergent adverse event

**Table 3:** Treatment-related treatment-emergent adverse events (all)

Number of events (%)^a^	Vamifeport QD (*n* = 9)	Vamifeport BID (*n* = 12)	Placebo (*n* = 4)
Anemia	1 (11.1)	1 (8.3)	0 (0)
Hemolysis	1 (11.1)	0 (0)	0 (0)
Discolored feces	0 (0)	1 (8.3)	0 (0)
Nausea	0 (0)	1 (8.3)	0 (0)
Vomiting	0 (0)	1 (8.3)	0 (0)
Fatigue	0 (0)	1 (8.3)	0 (0)
Non-cardiac chest pain	0 (0)	0 (0)	1 (25.0)
Pyrexia	0 (0)	1 (8.3)	0 (0)
Increased ALT	0 (0)	1 (8.3)	0 (0)
Increased AST	0 (0)	1 (8.3)	0 (0)
Increased CPK	0 (0)	1 (8.3)	0 (0)
Decreased appetite	0 (0)	1 (8.3)	0 (0)
Headache	0 (0)	1 (8.3)	1 (25.0)
Pollakiuria	0 (0)	1 (8.3)	0 (0)
Pruritus	0 (0)	0 (0)	1 (25.0)

^a^Each patient could experience > 1 treatment-related adverse event

ALT, alanine aminotransferase; AST, aspartate aminotransferase; BID, twice daily; CPK, creatine phosphokinase; QD, once daily

### Iron-related parameters

**Fig. 2:**
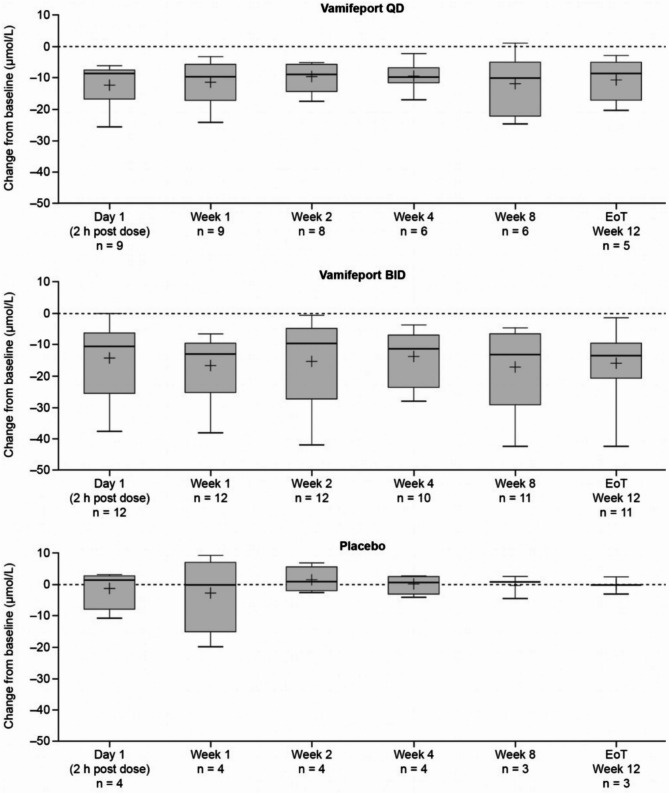
Box plots of mean (SD) change from baseline in total serum iron, over the 12-week treatment period (full analysis set, *N* = 25). BID, twice daily; EoT, end of treatment; QD, once daily; SD, standard deviation

**Fig. 3:**
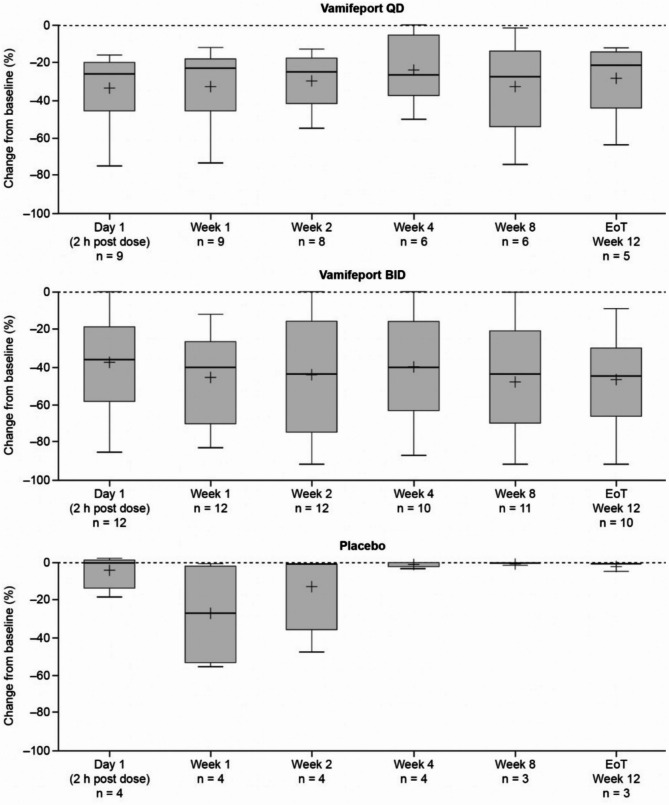
Box plots of mean (SD) change from baseline in TSAT, over the 12-week treatment period (full analysis set, *N* = 25). BID, twice daily; EoT, end of treatment; QD, once daily; SD, standard deviation; TSAT, transferrin saturation

**Fig. 4:**
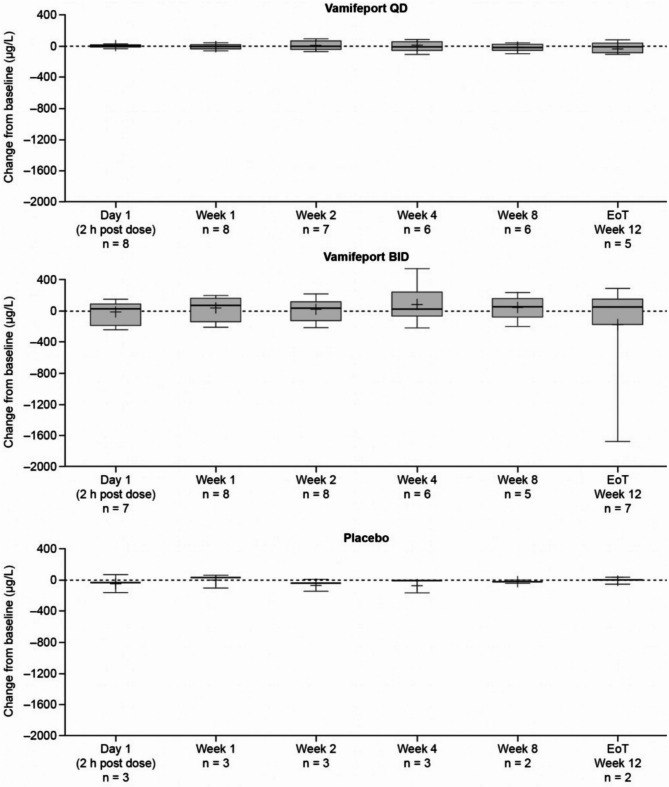
Box plots of mean (SD) change from baseline in serum ferritin levels, over the 12-week treatment period (full analysis set, *N* = 25). BID, twice daily; EoT, end of treatment; QD, once daily; SD, standard deviation

Mean total serum iron concentrations were similar at baseline across treatment groups and had already decreased by 2 h after vamifeport administration on Day 1 and Week 1 in all vamifeport-treated patients (Fig. 2). Mean (standard deviation [SD]) decreases of − 12.2 (6.5) and − 11.3 (7.2) µmol/L in vamifeport QD and − 14.5 (12.1) and − 17.0 (9.6) µmol/L in vamifeport BID occurred on Day 1 and Week 1, respectively. At each visit throughout the remaining 12-week treatment period, mean total serum iron levels were below baseline levels 2 h after vamifeport administration. Serum iron concentrations in the vamifeport BID group were, on average, maintained at a lower level than those in the vamifeport QD group throughout the 12-week treatment period, suggesting a dose-dependent effect. Summary statistics for serum iron concentrations over time are included in Supplemental Table 1. Mean baseline TSAT levels were also similar across treatment groups and had decreased by 2 h post vamifeport dose on Day 1 and Week 1 (Fig. 3) (mean [standard deviation] decrease: vamifeport QD − 33.6 [18.9] % and − 32.6 [19.6] %; vamifeport BID − 37.2 [27.6] % and − 44.8 [24.6] %). Mean TSAT remained below baseline levels 2 h post-dose at each subsequent visit in the vamifeport treatment groups. TSAT levels in the vamifeport BID group averaged at a lower level than those observed in the vamifeport QD group throughout the study. Summary statistics for TSAT levels over time are included in Supplemental Table 2; individual patient plots of TSAT% over time by treatment are included in Supplemental Fig. 2. There were also no meaningful changes in serum ferritin (Fig. 4; Supplemental Table 3), hepcidin levels (Supplemental Fig. 1; Supplemental Table 4), or LIC (data not shown) in any treatment group.

### Hematological parameters

**Fig. 5:**
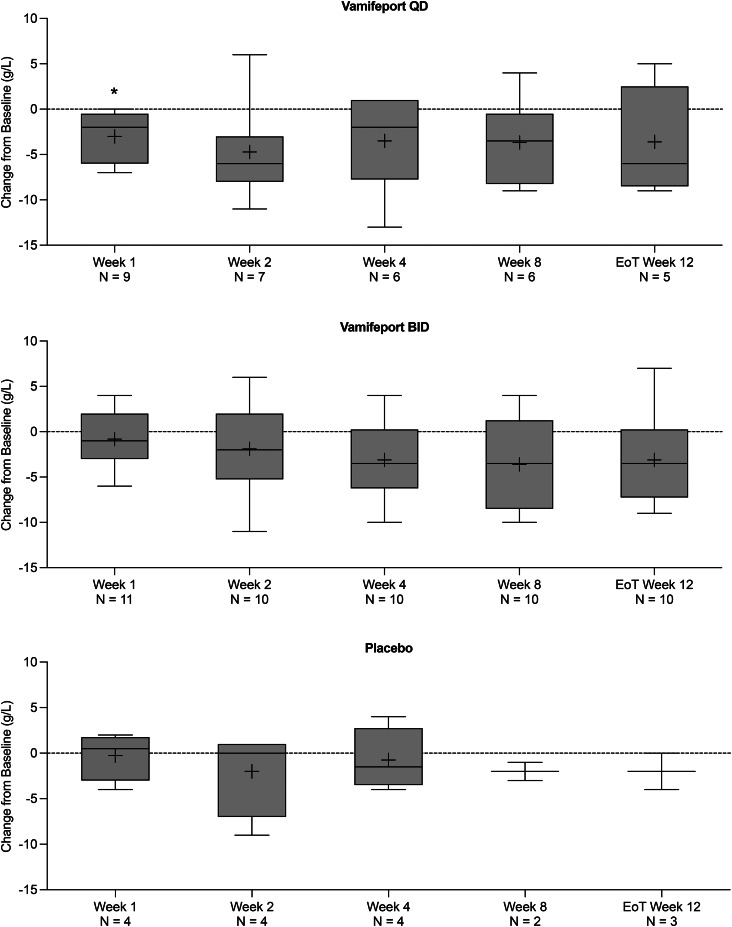
Box plots of mean (SD) change from baseline in serum hemoglobin levels, over the 12-week treatment period (full analysis set, *N* = 25). BID, twice daily; EoT, end of treatment; QD, once daily; SD, standard deviation. * Denotes a p-value < 0.05 in Dunnett’s multiple comparisons test

**Fig. 6:**
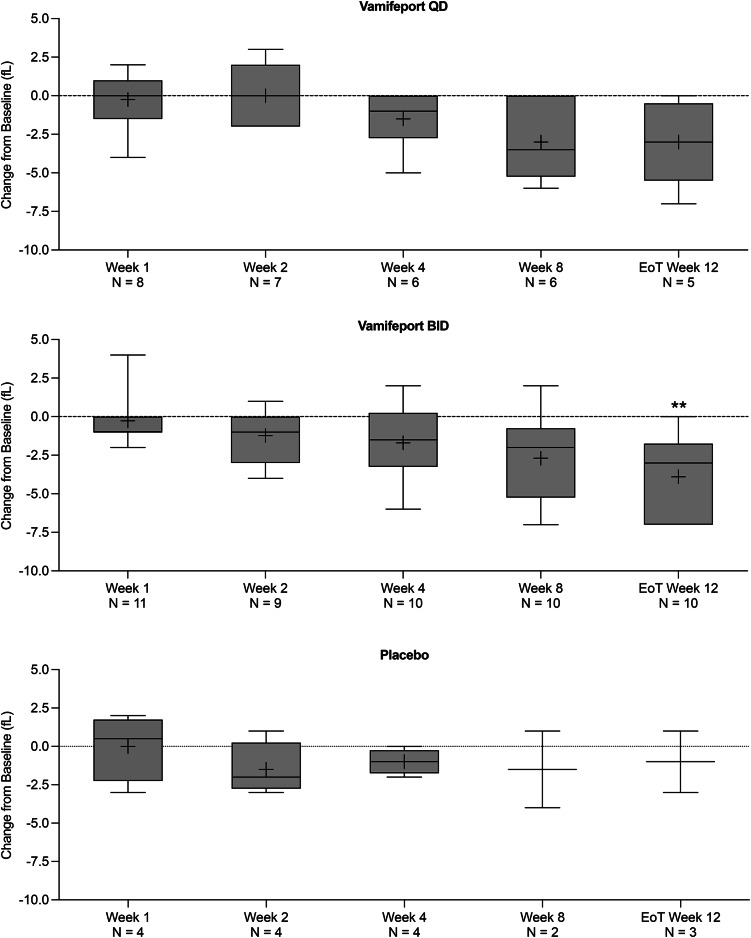
Box plots of mean (SD) change from baseline in MCV, over the 12-week treatment period (full analysis set, *N* = 25). BID, twice daily; EoT, end of treatment; MCV, mean corpuscular volume; QD, once daily; SD, standard deviation. ** Denotes a p-value < 0.01 in Dunnett’s multiple comparisons test

There was a downward trend in hemoglobin levels over time in patients receiving vamifeport (Fig. 5). Except for two patients who received a transfusion and were excluded from the final analysis of hemoglobin data, no patient experienced a change in hemoglobin levels ≥ 1.0 g/dL over the 12-week treatment period. Summary statistics for serum hemoglobin levels over time are included in Supplemental Table 5. A downward trend was also observed in MCV in patients receiving vamifeport QD and those receiving vamifeport BID (Fig. 6); this trend persisted up to two weeks (Week 12, EoT) after the last dose (*p* < 0.01). Summary statistics for MCV over time are included in Supplemental Table 6.

### Hemolytic and erythropoietic markers

**Fig. 7:**
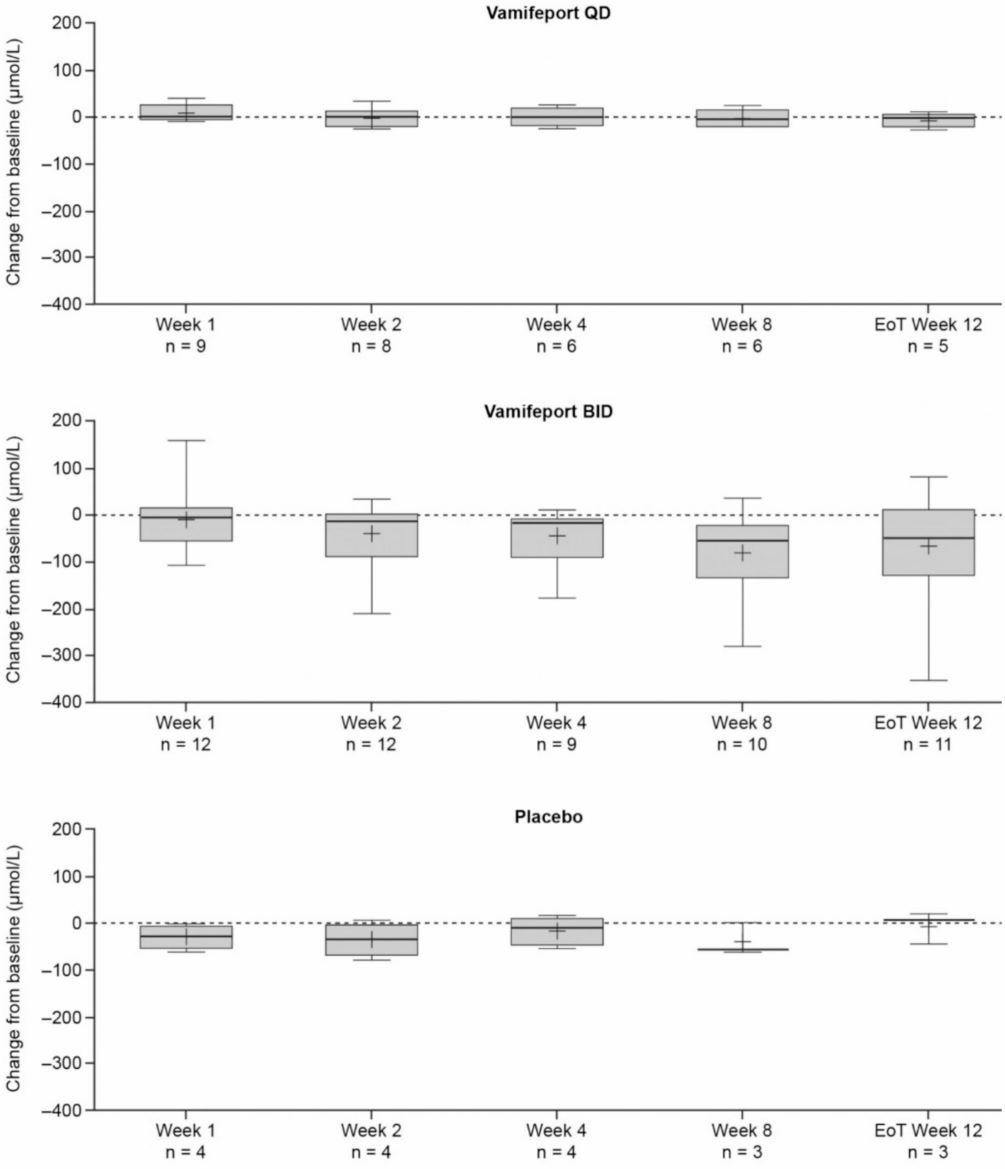
Box plots of mean (SD) change from baseline in LDH, over the 12-week treatment period (full analysis set, *N* = 25). BID, twice daily; EoT, end of treatment; LDH, lactate dehydrogenase; QD, once daily; SD, standard deviation

**Fig. 8:**
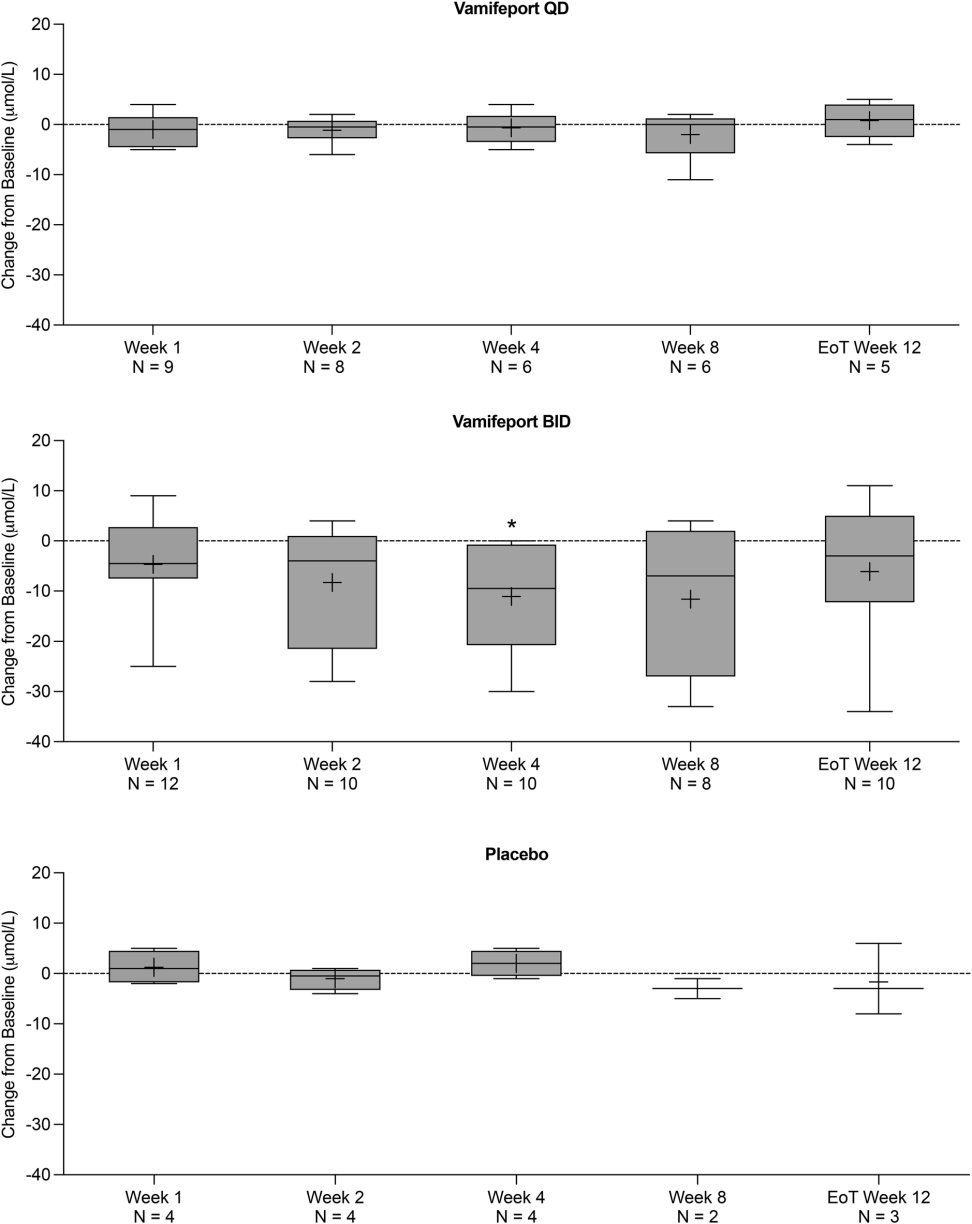
Box plots of mean (SD) change from baseline in unconjugated bilirubin levels, over the 12-week treatment period (full analysis set, *N* = 25). BID, twice daily; EoT, end of treatment; QD, once daily; SD, standard deviation. * Denotes a p-value < 0.05 in Dunnett’s multiple comparisons test

A wider range of LDH (Fig. 7) and unconjugated bilirubin (Fig. 8) levels at baseline were observed in the vamifeport BID group compared with the other treatment groups. Overall, no clinically relevant changes in hemolytic markers occurred over the 12-week study period in any treatment group. However, slight downward trends in LDH and unconjugated bilirubin levels over time were observed in some individuals during vamifeport BID treatment (Fig. 8), suggesting some improvements in the patients’ hemolysis activity. Summary statistics for LDH and unconjugated bilirubin over time are included in Supplemental Tables 7 and 8, respectively. Haptoglobin (data not shown) and fetal hemoglobin (Supplemental Fig. 3) levels were stable throughout the study. RBC and reticulocyte counts were also stable throughout the study (Supplemental Figs. 4‒7).

## Discussion

Overall, the results of this Phase 2a study show that oral vamifeport at doses of 60 or 120 mg QD or BID had a favorable safety and tolerability profile versus placebo in adults with β-NTDT. All reported TEAEs were mild or moderate in intensity, and no clinically meaningful changes were noted in laboratory values, vital signs, electrocardiogram readings, or physical examinations. Preliminary efficacy and promising target engagement in this patient population were also demonstrated in terms of the observed acute pharmacodynamic effects of vamifeport on total serum iron and TSAT levels.

The observed safety and tolerability results reported here are consistent with those observed in the Phase 1 study of vamifeport in healthy volunteers [9]. Oral vamifeport administered QD or BID also led to rapid and consistent decreases in total serum iron concentrations, with the most considerable reductions seen in the BID group; as expected, placebo had no clinically meaningful effect on serum iron levels. The pharmacodynamic effect of vamifeport on serum iron concentrations was as expected based on its mode of action. It demonstrated target engagement in this patient population, in line with observations in the Hbbth3/+ mouse model of NTDT [7] and the Phase 1 study of vamifeport in healthy volunteers [9]. Both vamifeport dosing schedules also rapidly and consistently reduced TSAT levels in patients with β-NTDT, similar to observations in the healthy volunteer study [9]. Mean baseline TSAT levels in the present study were 76.2%, which is above the 75% level when NTBI is detected [12]. TSAT levels were reduced by nearly one-third within 2 h of vamifeport QD or BID administration and maintained below baseline levels for the remainder of this study. The observed reduction in TSAT is important as previous studies have shown that elevated NTBI levels are associated with damage to the liver and heart [13, 14]. There were no clinically relevant changes in serum ferritin concentrations in the present study, as was observed previously in healthy volunteers [9]. Hepcidin concentrations were also unaffected by vamifeport treatment in our study, suggesting that hepcidin synthesis might be uncoupled from circulating and tissue iron levels in patients with β-thalassemia, in line with observations in a mouse model of NTDT [7]. In contrast, after a temporary increase, there was a general reduction in serum hepcidin concentrations over 7 days of vamifeport treatment in the study that included healthy volunteers [9].

Vamifeport treatment did not meaningfully improve markers of ineffective erythropoiesis in the present study, possibly due to the relatively low to moderate level of iron overload present in these patients at baseline. It is possible that vamifeport may be most efficacious in patients with high levels of iron overload or those with a younger age and a less compromised bone marrow microenvironment [15, 16]. Such patients may represent a more sensitive population to vamifeport and should be confirmed in future trials. A potentially interesting progressive downward trends were observed in MCV and for 2 out of 3 hemolytic markers (LDH and unconjugated bilirubin) in those receiving vamifeport, which reached a maximum at around Week 8. This suggests that iron restriction may, at least initially, decrease erythropoiesis in patients with β-thalassemia, as seen by a reduction in the MCV over time. However, in the long term, improvements in ineffective erythropoiesis may occur with a resultant progressive improvement in the quality of the patients’ RBCs over time, as the proportion that is produced under iron-restricted conditions increases [6]. These pharmacodynamic effects were mainly seen with vamifeport BID, suggesting that for optimum effects, continuous restriction of iron to the erythron is important. Nonetheless, these results remain inconclusive as haptoglobin levels were unchanged at 12 weeks.

Given the mechanism of action of vamifeport, suppression of hemoglobin synthesis would be an expected dose-dependent adverse effect. The downward trend in hemoglobin levels during vamifeport BID treatment is consistent with the similar trend in MCV noted in the present study. MCV also reduced during vamifeport treatment in a preclinical study of the effects of vamifeport in the Hbbth3/+ mouse model of β-thalassemia. However, hemoglobin levels increased after approximately one week of vamifeport treatment in that study [7]. The lack of a delayed hemoglobin increase (no patient experienced a hemoglobin increase ≥ 1 g/dL) and the trend towards reduced hemolytic marker levels in the present 12-week study may suggest that decreasing hemoglobin synthesis via ferroportin inhibition decreases hemolysis. This may be caused by the decrease in free α-globin chains due to iron restriction, as seen in severely iron-deficient β-thalassemia patients [17]. Fewer free α-globin chains lead to a decrease in inclusions in RBCs, as shown by Han et al. (2005) in a β-thalassemia mouse model, which underpins the therapeutic benefits of the iron-restriction hypothesis [18]. In contrast, in mice, the benefit of reducing hemolysis in the context of ineffective erythropoiesis may outweigh the inhibitory effects on hemoglobin synthesis. Nonetheless, the absence of increased rates of anemia in the present study suggests that evaluating the activity of higher vamifeport doses, increased dose frequencies, or more prolonged dosing (> 12 weeks) may be a feasible option in future studies in patients with β-NTDT. The favorable safety profile and novel mode of action of vamifeport also support the evaluation of this ferroportin inhibitor in combination with or sequential to other agents used to treat this condition.

### Limitations

A limitation of this Phase 2a study is that patient numbers were small. There was also a moderately high heterogeneity in some of the clinical and hematological characteristics of those included, as is common in studies of rare diseases. For example, proportionally more patients in the placebo group had received prior ICT at baseline than the vamifeport BID group, and proportionally more patients in the placebo group had received blood transfusion, splenectomy, or ongoing hydroxyurea treatment than the vamifeport QD and BID groups. Certain variability of baseline factors might have impacted the pharmacodynamic findings reported here. For example, some patients in this study had relative fetal hemoglobin expression levels of ~ 100% at baseline, which would hamper any relative increase in this parameter in these patients. The length of the study (12 weeks) could also have been a factor in the observed hemoglobin results. However, as noted above, hemoglobin increases have been observed in mouse models after approximately 1 week of vamifeport treatment [7]. It is also possible that patients with β-thalassemia do not respond hematologically to iron restriction. It would be interesting to see if the pharmacodynamic findings in the present study are confirmed in larger, more extended studies in a more homogeneous population of patients with β-NTDT. Given the reduction in TSAT levels observed here, it would also be interesting to evaluate the effect of vamifeport on serum NTBI levels in future studies. The inclusion of additional time points for biomarker assessment would also be helpful to gain more clarity on the time course of pharmacodynamic effects of vamifeport in patients with β-thalassemia. Finally, the impact of different patient baseline characteristics on the pharmacodynamics of vamifeport is also worthy of further investigation.

The results of this study highlight a potential concern in translating observations in animal β-thalassemia models to patients – observations in animal models of this disease have not consistently been reproduced in patients with β-thalassemia. For example, in the Hbbth3/+ mouse model of β-thalassemia, the observed effects of vamifeport on iron-restricted erythropoiesis seem to be mediated by the spleen and are extramedullary [7], which is not the case in humans. Thus, in thallasemic patients, a longer period of exposure to vamifeport may be required to revoke the long-standing iron-induced toxicity to the bone marrow milieu, before observing improvements in hematological parameters. Nonetheless, ferroportin inhibition would be expected to reduce iron overload and the resultant organ toxicity in a clinical setting in β-thalassemia and other iron-loading diseases. Similar issues with conflicting results observed in preclinical and clinical settings have been reported for other investigative treatments for β-thalassemia, including those for bitopertin [19, 20], rusfertide (PTG-300) [4, 21], and LJPC-401 [22–24]. Although our knowledge of the pathophysiology of β-thalassemia has increased over recent years, these observations suggest that we do not yet have a complete understanding of this condition. Therefore, improved animal models may be required to better reflect the human pathophysiology of β-thalassemia. Nonetheless, the pharmacodynamic effects of vamifeport observed in the present study concerning total serum iron and TSAT levels align with findings in preclinical models and studies in healthy volunteers. The study demonstrates promising target engagement, with vamifeport blocking the efflux of iron into the circulation via ferroportin.

## Conclusions

In summary, in this 12-week Phase 2a study in adults with b-NTDT, oral vamifeport at doses up to 120 mg BID had a favorable safety and tolerability profile and showed promising target engagement and pharmacodynamic effects on total serum iron and TSAT levels versus placebo. However, the efficacy of vamifeport in patients with β-NTDT will need to be explored through additional studies, and the optimum vamifeport dose and schedule also remain to be determined. Limiting the availability of iron for erythropoiesis through ferroportin inhibition might be a possible treatment approach for patients with β-NTDT, as well as those with other conditions associated with ineffective erythropoiesis or iron overload such as sickle cell disease, polycythemia vera, and hemochromatosis. However, the results from further studies are awaited.

## Supplementary Information

Below is the link to the electronic supplementary material.

Supplementary Material 1

## Data Availability

The datasets supporting the conclusions of this article are included within the article and its additional file. CSL will consider on a case-by-case basis requests to share Individual Patient Data (IPD) with external bona-fide, qualified scientific and medical researchers. For information on the process and requirements for submitting a voluntary data sharing request for IPD, please contact CSL at clinicaltrials@cslbehring.com.

## Abbreviations

ALT: Alanine aminotransferase
AST: Aspartate aminotransferase
β-NTDT: Non-transfusion-dependent beta-thalassemia
BID: Twice daily
ChfB: Change from baseline
CPK: Creatine phosphokinase
EOT: End of treatment
FAS: Full analysis set
Hb: Hemoglobin
HU: Hydroxyurea
ICT: Iron chelation therapy
LDH: Lactate dehydrogenase
LIC: Liver iron content
MCV: Mean corpuscular volume
NTBI: Non-transferrin bound iron
NTDT: Non-transfusion-dependent thalassemia
PPS: Per-protocol set
Q1: First quartile
Q3: Third quartile
QD: Once daily
RBC: Red blood cells
SD: Standard deviation
SS: Safety set
TEAE: Treatment-emergent adverse event
TSAT: Transferrin saturation
VIT-2763: Vamifeport
