# Supplementary material for: Safety and pharmacodynamics of the ferroportin inhibitor vamifeport in patients with non-transfusion-dependent β-thalassemia: results from a randomized phase 2a study
Source: Orphanet J Rare Dis. 2025 Nov 25;20:608. doi: 10.1186/s13023-025-04119-y (PMC12648932; doi:10.1186/s13023-025-04119-y)
Supplement: Supplementary file 1 — Supplementary Material 1 [file 13023_2025_4119_MOESM1_ESM.docx]

## Supplemental Table 1. Summary statistics for total serum iron (µmol/L) over time (full analysis set, N=25).^a^

|  | Vamifeport QD (n=9) | | Vamifeport BID (n=12) | | Placebo (n=4) | |
| --- | --- | --- | --- | --- | --- | --- |
|  | Value | ChfB | Value | ChfB | Value | ChfB |
| **Baseline^b^** | | | | | | |
| n | 9 |  | 12 |  | 4 |  |
| Mean (SD) | 23.8 (13.4) |  | 28.4 (9.8) |  | 30.9 (13.3) |  |
| Median | 17.0 |  | 29.7 |  | 34.5 |  |
| Q1, Q3 | 14.4, 38.4 |  | 19.3, 45.3 |  | 16.9, 41.3 |  |
| Min, max | 12.1, 46.5 |  | 14.8, 45.3 |  | 12.2, 42.3 |  |
| **Day 1^c^** | | | | | | |
| n | 9 | 9 | 12 | 12 | 4 | 4 |
| Mean (SD) | 11.6 (9.3) | −12.2 (6.5) | 14.0 (12.3) | −14.5 (12.1) | 29.8 (11.9) | −1.1 (6.5) |
| Median | 8.2 | −8.5 | 8.0 | −10.8 | 30.8 | 1.6 |
| Q1, Q3 | 6.0, 18.3 | −17.0, −7.6 | 5.7, 23.5 | −25.7, −6.6 | 17.9, 40.7 | −7.9, 3.0 |
| Min, max | 3.7, 24.8 | −25.8, −6.0 | 2.1, 40.0 | −37.7, −0.2 | 14.7, 42.9 | −10.7, 3.2 |
| **Week 1^c^** | | | | | | |
| n | 9 | 9 | 12 | 12 | 4 | 4 |
| Mean (SD) | 12.5 (9.2) | −11.3 (7.2) | 11.4 (7.7) | −17.0 (9.6) | 28.2 (17.9) | −2.7 (12.4) |
| Median | 9.7 | −9.7 | 9.1 | −13.1 | 25.1 | −0.1 |
| Q1, Q3 | 6.5, 18.1 | −17.3, −5.6 | 7.0, 11.8 | −25.1, −9.6 | 12.8, 46.8 | −15.3, 7.3 |
| Min, max | 4.2, 32.3 | −24.3, −3.1 | 5.3, 33.3 | −38.0, −6.9 | 10.9, 51.7 | −19.9, 9.4 |
| **Week 2^c^** | | | | | | |
| n | 8 | 8 | 12 | 12 | 4 | 4 |
| Mean (SD) | 11.7 (8.6) | −9.7 (4.7) | 13.0 (9.0) | −15.4 (12.8) | 32.5 (15.5) | 1.6 (4.0) |
| Median | 9.2 | −8.8 | 11.6 | −9.6 | 34.6 | 1.0 |
| Q1, Q3 | 6.0, 15.2 | −14.4, −5.6 | 6.5, 16.2 | −27.3, −5.0 | 17.3, 45.8 | −1.9, 5.7 |
| Min, max | 4.1, 30.9 | −17.5, −4.9 | 3.7, 36.1 | −41.6, −0.8 | 11.6, 49.1 | −2.3, 6.8 |
| **Week 4^c^** | | | | | | |
| n | 6 | 6 | 10 | 10 | 4 | 4 |
| Mean (SD) | 13.9 (12.2) | −9.3 (4.7) | 12.9 (8.0) | −14.1 (8.9) | 31.1 (13.7) | 0.2 (2.9) |
| Median | 10.4 | −9.5 | 9.5 | −11.5 | 33.4 | 0.9 |
| Q1, Q3 | 4.5, 22.0 | −11.5, −6.7 | 7.3, 18.3 | −23.9, −7.2 | 17.3, 42.5 | −2.8, 2.6 |
| Min, max | 4.0, 36.8 | −16.9, −2.3 | 4.9, 30.8 | −27.9, −3.7 | 12.3, 45.2 | −3.8, 2.9 |
| **Week 8^c^** | | | | | | |
| n | 6 | 6 | 11 | 11 | 3 | 3 |
| Mean (SD) | 11.4 (7.8) | −11.9 (9.6) | 11.6 (9.7) | −17.1 (11.9) | 26.7 (12.0) | −0.4 (3.6) |
| Median | 8.5 | −9.9 | 9.3 | −13.3 | 33.4 | 0.6 |
| Q1, Q3 | 5.4, 17.8 | −22.0, −4.9 | 5.3, 17.8 | −29.3, −7.0 | 12.8, 33.9 | −4.3, 2.6 |
| Min, max | 4.9, 25.4 | −24.7, 1.0 | 3.0, 35.3 | −42.3, −4.9 | 12.8, 33.9 | −4.3, 2.6 |
| **EOT Week 12^c^** | | | | | | |
| n | 5 | 5 | 11 | 11 | 3 | 3 |
| Mean (SD) | 13.7 (11.0) | −10.5 (6.8) | 11.7 (6.1) | −16.0 (10.4) | 27.1 (12.9) | 0.03 (2.7) |
| Median | 11.4 | −8.5 | 10.4 | −13.8 | 33.5 | 0.1 |
| Q1, Q3 | 5.6, 23.0 | −17.3, −4.9 | 5.7, 18.2 | −21.1, −9.7 | 12.3, 35.5 | −2.7, 2.7 |
| Min, max | 3.6, 32.2 | −20.2, −2.9 | 3.4, 23.3 | −41.9, −1.4 | 12.3, 35.5 | −2.7, 2.7 |

^a^Laboratory values expressed as “less than” or “greater than” have been imputed using the next numerical value. Patients who received a blood transfusion were censored from their first transfusion date; ^b^Baseline values based on samples taken at screening; ^c^Values based on samples taken 2 h post dose.

BID, twice daily; ChfB, change from baseline; EOT, end of treatment; QD, once daily; Q1, 1^st^ quartile; Q3, 3rd quartile; SD, standard deviation.

## Supplemental Table 2. Summary statistics for TSAT (%) over time (full analysis set, N=25).^a^

|  | Vamifeport QD (n=9) | | Vamifeport BID (n=12) | | Placebo (n=4) | |
| --- | --- | --- | --- | --- | --- | --- |
|  | Value | ChfB | Value | ChfB | Value | ChfB |
| **Baseline^b^** | | | | | | |
| n | 9 |  | 12 |  | 4 |  |
| Mean (SD) | 69.3 (31.2) |  | 79.0 (24.1) |  | 83.3 (33.5) |  |
| Median | 72.0 |  | 89.5 |  | 100.0 |  |
| Q1, Q3 | 36.5, 100.0 |  | 53.3, 100.0 |  | 49.8, 100.0 |  |
| Min, max | 34.0, 100.0 |  | 44.0, 100.0 |  | 33.0, 100.0 |  |
| **Day 1^c^** | | | | | | |
| n | 9 | 9 | 12 | 12 | 4 | 4 |
| Mean (SD) | 35.8 (24.3) | −33.6 (18.9) | 41.83 (34.2) | −37.2 (27.6) | 79.3 (30.7) | −4.0 (9.4) |
| Median | 27.0 | −26.0 | 23.5 | −36.0 | 91.0 | 0.0 |
| Q1, Q3 | 16.0, 62.5 | −45.5, −20.0 | 15.3, 70.8 | −58.5, −18.5 | 46.8, 100.0 | −13.5, 1.5 |
| Min, max | 10.0, 74.0 | −75.0, −16.0 | 6.0, 100.0 | −85.0, 0.0 | 35.0, 100.0 | −18.0, 2.0 |
| **Week 1^c^** | | | | | | |
| n | 9 | 9 | 12 | 12 | 4 | 4 |
| Mean (SD) | 36.8 (24.4) | −32.6 (19.6) | 34.3 (20.9) | −44.8 (24.6) | 56.5 (30.6) | −26.8 (28.8) |
| Median | 30.0 | −23.0 | 24.0 | −40.0 | 48.5 | −26.0 |
| Q1, Q3 | 15.5, 57.5 | −46.0, −18.0 | 19.3, 47.0 | −69.8, −27.0 | 33.0, 88.0 | −53.3, −1.0 |
| Min, max | 12, 83 | −73.0, −12.0 | 16.0, 86.0 | −83.0, −12.0 | 29.0, 100.0 | −55.0, 0.0 |
| **Week 2^c^** | | | | | | |
| n | 8 | 8 | 12 | 12 | 4 | 4 |
| Mean (SD) | 36.0 (22.8) | −29.5 (14.6) | 34.8 (24.1) | −43.2 (33.2) | 71.5 (33.9) | −11.8 (23.5) |
| Median | 28.5 | −25.0 | 34.5 | −43.5 | 76.5 | 0.0 |
| Q1, Q3 | 16.0, 58.5 | −42.3, −17.3 | 19.3, 42.0 | −77.0, −15.8 | 38.0, 100.0 | −35.3, 0.0 |
| Min, max | 11, 74 | −55.0, −13.0 | 9.0, 100.00 | −91.0, 0.0 | 33.0, 100.0 | −47.0, 0.0 |
| **Week 4^c^** | | | | | | |
| n | 6 | 6 | 10 | 10 | 4 | 4 |
| Mean (SD) | 41.0 (32.3) | −24.0 (18.2) | 39.3 (26.0) | −40.2 (28.6) | 82.5 (35.0) | −0.8 (1.5) |
| Median | 34.5 | −26.5 | 34.5 | −40.0 | 100.0 | 0.0 |
| Q1, Q3 | 15.3, 62.5 | −38.0, −5.3 | 19.5, 54.0 | −63.3, −15.8 | 47.5, 100.0 | −2.3, 0.0 |
| Min, max | 10, 100 | −50.0, 0.0 | 13.0, 100.0 | −87.0, 0.0 | 30.0, 100.0 | −3.0, 0.0 |
| **Week 8^c^** | | | | | | |
| n | 6 | 6 | 11 | 11 | 3 | 3 |
| Mean (SD) | 32.3 (17.5) | −32.7 (25.8) | 33.5 (27.1) | −47.9 (29.7) | 77.3 (39.3) | −0.3 (0.6) |
| Median | 26.5 | −27.5 | 28.0 | −44.0 | 100.0 | 0.0 |
| Q1, Q3 | 21.8, 44.0 | −54.5, −13.8 | 12.0, 52.0 | −70.0, −20.0 | 32.0, 100.0 | −1.0, 0.0 |
| Min, max | 15.0, 65.0 | −74.0, −1.0 | 8.0, 100.0 | −92.0, 0.0 | 32, 100 | −1.0, 0.0 |
| **EOT Week 12^c^** | | | | | | |
| n | 5 | 5 | 10 | 10 | 3 | 3 |
| Mean (SD) | 36.0 (25.9) | −27.6 (20.4) | 34.8 (17.8) | −46.8 (23.6) | 76.3 (41.0) | −1.3 (2.3) |
| Median | 28.0 | −21.0 | 33.5 | −45.0 | 100.0 | 0.0 |
| Q1, Q3 | 18.0, 58.0 | −−44.0, −14.5 | 15.8, 48.5 | −66.3, −29.5 | 29.0, 100.0 | −4.0, 0.0 |
| Min, max | 10.0, 79.0 | −63.0, −12.0 | 9.0, 65.0 | −91.0, −9.0 | 29, 100 | −4.0, 0.0 |

^a^Laboratory values expressed as “less than” or “greater than” have been imputed using the next numerical value. Patients who received a blood transfusion were censored from their first transfusion date; ^b^Baseline values based on samples taken at screening; ^c^Values based on samples taken 2 h post dose.

BID, twice daily; ChfB, change from baseline; EOT, end of treatment; QD, once daily; Q1, 1^st^ quartile; Q3, 3rd quartile; SD, standard deviation; TSAT, transferrin saturation.

## Supplemental Table 3. Summary statistics for serum ferritin (µg/L) over time (full analysis set, N=25).^a^

|  | Vamifeport QD (n=9) | | Vamifeport BID (n=12) | | Placebo (n=4) | |
| --- | --- | --- | --- | --- | --- | --- |
|  | Value | ChfB | Value | ChfB | Value | ChfB |
| **Baseline^b^** | | | | | | |
| n | 8 |  | 9 |  | 3 |  |
| Mean (SD) | 426.9 (229.6) |  | 1133.2 (2119.1) |  | 440.0 (321.5) |  |
| Median | 374.8 |  | 442.1 |  | 257.4 |  |
| Q1, Q3 | 284.3, 446.0 |  | 266.3, 724.2 |  | 251.5, 811.2 |  |
| Min, max | 213.1, 953.6 |  | 132.0, 6754.0 |  | 251.5, 811.2 |  |
| **Day 1^c^** | | | | | | |
| n | 8 | 8 | 9 | 7 | 4 | 3 |
| Mean (SD) | 431.9 (226.0) | 4.26 (15.2) | 445.3 (194.1) | −17.7 (139.3) | 367.6 (208.7) | −24.1 (113.3) |
| Median | 398.5 | 4.4 | 450.3 | 34.5 | 288.9 | −16.3 |
| Q1, Q3 | 292.5, 465.9 | −8.8, 17.2 | 257.2, 594.9 | −175.7, 91.2 | 225.8, 588.2 | −141.1, 85.10 |
| Min, max | 194.2, 941.8 | −18.9, 27.3 | 227.7, 799.6 | −230.5, 148.9 | 222.7, 670.1 | −141.1, 85.10 |
| **Week 1^c^** | | | | | | |
| n | 8 | 8 | 8 | 8 | 3 | 3 |
| Mean (SD) | 416.8 (220.6) | −10.1 (31.5) | 471.5 (209.8) | 40.9 (164.5) | 445.8 (228.2) | 5.8 (94.0) |
| Median | 359.0 | −4.8 | 472.9 | 75.2 | 321.9 | 48.9 |
| Q1, Q3 | 286.6, 470.9 | −32.7, 19.5 | 266.5, 600.0 | −140.9, 174.4 | 306.3, 709.2 | −102.0, 70.4 |
| Min, max | 209.0, 920.8 | −63.3, 30.4 | 198.6, 832.2 | −214.1, 214.7 | 306.3, 709.2 | −102.0, 70.4 |
| **Week 2^c^** | | | | | | |
| n | 7 | 7 | 9 | 8 | 4 | 3 |
| Mean (SD) | 441.3 (269.6) | 3.3 (60.6) | 464.1 (210.8) | 23.8 (149.1) | 411.4 (207.5) | −49.8 (76.9) |
| Median | 372.7 | −8.8 | 486.4 | 44.5 | 370.8 | −30.1 |
| Q1, Q3 | 296.2, 436.7 | −52.4, 73.2 | 309.2, 563.1 | −123.1, 126.8 | 237.2, 626.1 | −134.6, 15.3 |
| Min, max | 206.3, 1026.8 | −67.7, 95.8 | 160.0, 877.8 | −213.1, 227.1 | 227.3, 676.6 | −134.6, 15.3 |
| **Week 4^c^** | | | | | | |
| n | 6 | 6 | 7 | 6 | 3 | 3 |
| Mean (SD) | 476.6 (282.5) | 1.1 (61.6) | 577.3 (335.2) | 90.0 (249.4) | 389.8 (240.6) | −50.2 (81.0) |
| Median | 375.8 | −5.8 | 590.0 | 39.7 | 251.4 | −6.9 |
| Q1, Q3 | 316.1, 602.5 | −47.6, 59.3 | 230.8, 799.4 | −66.6, 246.3 | 250.5, 667.6 | −143.6, −0.1 |
| Min, max | 284.1, 1040.9 | −83.7, 87.3 | 166.2, 1118.6 | −207.7, 539.2 | 250.5, 667.6 | −143.6, −0.1 |
| **Week 8^c^** | | | | | | |
| n | 6 | 6 | 7 | 5 | 3 | 2 |
| Mean (SD) | 463.7 (206.6) | −11.9 (43.1) | 508.6 (211.4) | 52.8 (158.0) | 340.2 (165.4) | −9.5 (20.6) |
| Median | 416.1 | −5.2 | 498.9 | 56.8 | 256.6 | −9.5 |
| Q1, Q3 | 323.3, 544.6 | −49.1, 24.2 | 371.3, 599.2 | −70.4, 174.1 | 233.4, 530.7 | −24.0, 5.1 |
| Min, max | 316.5, 872.3 | −81.3, 39.6 | 238.4, 894.4 | −193.5, 243.7 | 233.4, 530.7 | −24.0, 5.1 |
| **EOT Week 12^c^** | | | | | | |
| n | 5 | 5 | 9 | 7 | 2 | 2 |
| Mean (SD) | 465.7 (227.4) | −15.8 (66.2) | 978.9 (1558.6) | −169.6 (670.2) | 261.7 (53.0) | 7.3 (57.2) |
| Median | 396.2 | −4.5 | 418.9 | 70.3 | 261.7 | 7.3 |
| Q1, Q3 | 332.4, 633.9 | −78.0, 40.7 | 310.1, 766.6 | −163.8, 152.1 | 224.2, 299.2 | −33.2, 47.7 |
| Min, max | 307.5, 866.9 | −86.7, 80.3 | 202.3, 5095.0 | −1659.0, 282.5 | 224.2, 299.2 | −33.2, 47.7 |

^a^Laboratory values expressed as “less than” or “greater than” have been imputed using the next numerical value. Patients who received a blood transfusion were censored from their first transfusion date; ^b^Baseline values based on samples taken at screening; ^c^Values based on samples taken 2 h post dose.

BID, twice daily; ChfB, change from baseline; EOT, end of treatment; QD, once daily; Q1, 1^st^ quartile; Q3, 3rd quartile; SD, standard deviation.

## Supplemental Table 4. Summary statistics for hepcidin (nmol/L) over time (full analysis set, N=25).^a^

|  | Vamifeport QD (n=9) | | Vamifeport BID (n=12) | | Placebo (n=4) | |
| --- | --- | --- | --- | --- | --- | --- |
|  | Value | ChfB | Value | ChfB | Value | ChfB |
| **Baseline^b^** | | | | | | |
| n | 9 |  | 12 |  | 4 |  |
| Mean (SD) | 5.7 (4.1) |  | 5.8 (6.4) |  | 2.3 (3.1) |  |
| Median | 4.2 |  | 4.8 |  | 1.2 |  |
| Q1, Q3 | 1.9, 9.8 |  | 1.1, 7.3 |  | 0.1, 5.6 |  |
| Min, max | 0.2, 11.0 |  | 0.2, 24.4 |  | 0.1, 6.8 |  |
| **Week 1^c^** | | | | | | |
| n | 9 | 9 | 12 | 12 | 4 | 4 |
| Mean (SD) | 4.2 (3.6) | −1.5 (2.6) | 3.5 (2.9) | −2.2 (4.7) | 2.2 (3.3) | −0.1 (0.6) |
| Median | 3.9 | −0.2 | 2.6 | −0.8 | 0.9 | 0.12 |
| Q1, Q3 | 0.7, 8.2 | −2.5, 0.0 | 1.1, 6.8 | −2.2, 0.0 | 0.2, 5.6 | −0.7, 0.3 |
| Min, max | 0.2, 9.3 | −7.8, 0.2 | 0.1, 8.5 | −15.9, 1.9 | 0.1, 7.1 | −1.0, 0.3 |
| **Week 2^c^** | | | | | | |
| n | 8 | 8 | 12 | 12 | 4 | 4 |
| Mean (SD) | 4.5 (3.0) | −1.8 (2.3) | 2.9 (2.5) | −2.9 (5.1) | 1.1 (1.7) | −1.3 (1.6) |
| Median | 4.3 | −1.2 | 2.1 | −1.0 | 0.3 | −0.9 |
| Q1, Q3 | 2.4, 6.0 | −4.5, −0.2 | 0.8, 6.0 | −3.5, −0.1 | 0.1, 2.8 | −2.9, 0.0 |
| Min, max | 0.3, 10 | −5.4, 1.0 | 0.1, 6.3 | −18.2, 0.6 | 0.1, 3.6 | −3.2, 0.0 |
| **Week 4^c^** | | | | | | |
| n | 6 | 6 | 11 | 11 | 4 | 4 |
| Mean (SD) | 5.9 (4.2) | −1.3 (3.7) | 3.5 (2.5) | −2.8 (5.4) | 1.4 (2.0) | −0.9 (1.2) |
| Median | 5.2 | −0.6 | 3.8 | −2.2 | 0.6 | −0.67 |
| Q1, Q3 | 3.5, 8.4 | −5.5, 1.2 | 0.9, 5.7 | −3.4, 0.0 | 0.2, 3.5 | −2.1, 0.1 |
| Min, max | 0.4, 13.0 | −5.7, 4.1 | 0.5, 8.0 | −18.1, 2.6 | 0.1, 4.5 | −2.4, 0.1 |
| **Week 8^c^** | | | | | | |
| n | 6 | 6 | 11 | 11 | 3 | 3 |
| Mean (SD) | 5.7 (3.8) | −1.5 (2.6) | 4.0 (4.3) | −1.8 (4.5) | 3.5 (5.3) | 0.42 (2.3) |
| Median | 5.6 | −1.8 | 1.8 | −0.2 | 0.5 | 0.1 |
| Q1, Q3 | 1.8, 9.7 | −3.9, 1.1 | 0.8, 8.5 | −3.0, 1.6 | 0.4, 9.7 | −1.7, 2.8 |
| Min, max | 1.1, 10.0 | −4.7, 2.0 | 0.1, 11.0 | −13.3, 2.5 | 0.4, 9.7 | −1.7, 2.8 |
| **EOT Week 12^c^** | | | | | | |
| n | 5 | 5 | 10 | 10 | 3 | 3 |
| Mean (SD) | 7.0 (5.4) | 0.5 (3.4) | 2.9 (3.2) | −2.7 (5.5) | 3.0 (4.3) | −0.1 (1.2) |
| Median | 7.0 | 0.3 | 1.8 | −1.0 | 0.9 | −0.16 |
| Q1, Q3 | 2.2, 12.0 | −2.2, 3.2 | 0.7, 4.0 | −3.2, −0.1 | 0.2, 8.0 | −1.3, 1.2 |
| Min, max | 0.5, 15.0 | −4.0, 5.6 | 0.1, 9.7 | −17.0, 2.9 | 0.2, 8.0 | −1.3, 1.2 |

^a^Laboratory values expressed as “less than” or “greater than” have been imputed using the next numerical value. Patients who received a blood transfusion were censored from their first transfusion date; ^b^Baseline values based on samples taken on Day 1, shortly before administration of first dose; ^c^Values based on samples taken 2 h post dose.

BID, twice daily; ChfB, change from baseline; EOT, end of treatment; QD, once daily; Q1, 1^st^ quartile; Q3, 3rd quartile; SD, standard deviation.

## Supplemental Table 5. Summary statistics for serum hemoglobin (g/L) over time (full analysis set, N=25).^a^

|  | Vamifeport QD (n=9) | | | Vamifeport BID (n=12) | | Placebo (n=4) | |
| --- | --- | --- | --- | --- | --- | --- | --- |
|  | Value | | ChfB | Value | ChfB | Value | ChfB |
| **Baseline^b^** | | | | | | | |
| n | 9 | |  | 12 |  | 4 |  |
| Mean (SD) | 88.2 (17.5) | |  | 88.5 (14.7) |  | 92.3 (9.8) |  |
| Median | 89.0 | |  | 88.0 |  | 91.5 |  |
| Q1, Q3 | 73.0, 106.5 | |  | 80.0, 99.5 |  | 83.5, 101.8 |  |
| Min, max | 63.0, 109.0 | |  | 61.0, 111.0 |  | 81.0, 105.0 |  |
| **Week 1^c^** | | | | | | | |
| n | 9 | 9 | | 11 | 11 | 4 | 4 |
| Mean (SD) | 85.2 (16.2) | −3.0 (2.7) | | 90.2 (13.0) | −0.8 (3.1) | 92.0 (11.1) | −0.3 (2.6) |
| Median | 84.0 | −2.0 | | 92.0 | −1.0 | 90.0 | 0.5 |
| Q1, Q3 | 70.5, 102.5 | −6.0, −0.5 | | 80.0, 101.0 | −3.0, 2.0 | 82.5, 103.5 | −3.0, 1.8 |
| Min, max | 62.0, 105.0 | −7.0, 0.0 | | 68.0, 108.0 | −6.0, 4.0 | 81.0, 107.0 | −4.0, 2.0 |
| **Week 2^c^** | | | | | | | |
| n | 7 | 7 | | 10 | 10 | 4 | 4 |
| Mean (SD) | 90.7 (14.0) | −4.7 (5.4) | | 87.6 (12.8) | −1.9 (4.8) | 90.3 (11.7) | −2.0 (4.8) |
| Median | 85.0 | −6.0 | | 89.5 | −2.0 | 87.5 | 0.0 |
| Q1, Q3 | 80.0, 103.0 | −8.0, −3.0 | | 76.3, 99.0 | −5.3, 2.0 | 80.8, 102.5 | −7.0, 1.0 |
| Min, max | 75, 111 | −11.0, 6.0 | | 68.0, 106.0 | −11.0, 6.0 | 80.0, 106.0 | −9.0, 1.0 |
| **Week 4^c^** | | | | | | | |
| n | 6 | 6 | | 10 | 10 | 4 | 4 |
| Mean (SD) | 92.7 (13.4) | −3.5 (5.5) | | 82.9 (11.6) | −3.1 (4.2) | 91.5 (8.5) | −0.8 (3.4) |
| Median | 92.5 | −2.0 | | 84.5 | −3.5 | 88.5 | −1.5 |
| Q1, Q3 | 79.0, 107.0 | −7.8, 1.0 | | 72.5, 89.0 | −6.3, 0.3 | 85.8, 100.3 | −3.5, 2.8 |
| Min, max | 77.0, 109.0 | −13.0, 1.0 | | 60.0, 101.0 | −10.0, 4.0 | 85.0, 104.0 | −4.0, 4.0 |
| **Week 8^c^** | | | | | | | |
| n | 6 | 6 | | 10 | 10 | 2 | 2 |
| Mean (SD) | 92.5 (14.0) | −3.7 (4.7) | | 81.5 (12.6) | −3.6 (5.0) | 84.5 (9.2) | −2.0 (1.4) |
| Median | 92.5 | −3.5 | | 85.0 | −3.5 | 84.5 | −2.0 |
| Q1, Q3 | 79.5, 106.0 | −8.3, −0.5 | | 71.3, 92.0 | −8.5, 1.3 | 78.0, 91.0 | −3.0, −1.0 |
| Min, max | 75.0, 109.0 | −9.0, 4.0 | | 60.0, 96.0 | −10.0, 4.0 | 78.0, 91.0 | −3.0, −1.0 |
| **EOT Week 12^c^** | | | | | | | |
| n | 5 | 5 | | 10 | 10 | 3 | 3 |
| Mean (SD) | 95.2 (13.6) | −3.6 (5.9) | | 88.2 (13.8) | −3.1 (5.2) | 90.7 (13.2) | −2.0 (2.0) |
| Median | 100.0 | −6.0 | | 89.0 | −3.5 | 88.0 | −2.0 |
| Q1, Q3 | 82.0, 106.0 | −8.5, 2.5 | | 76.8, 94.8 | −7.3, 0.25 | 79.0, 105.0 | −4.0, 0.0 |
| Min, max | 75.0, 110.0 | −9.0, 5.0 | | 69.0, 118.0 | −9.0, 7.0 | 79.0, 105.0 | −4.0, 0.0 |

^a^Laboratory values expressed as “less than” or “greater than” have been imputed using the next numerical value. Patients who received a blood transfusion were censored from their first transfusion date; ^b^Baseline values based on samples taken on Day 1, shortly before administration of first dose; ^c^Values based on samples taken 2 h post dose.

BID, twice daily; ChfB, change from baseline; EOT, end of treatment; QD, once daily; Q1, 1^st^ quartile; Q3, 3rd quartile; SD, standard deviation.

## Supplemental Table 6. Summary statistics for MCV (fL) over time (full analysis set, N=25).^a^

|  | Vamifeport QD (n=9) | | Vamifeport BID (n=12) | | Placebo (n=4) | |
| --- | --- | --- | --- | --- | --- | --- |
|  | Value | ChfB | Value | ChfB | Value | ChfB |
| **Baseline^b^** | | | | | | |
| n | 9 |  | 12 |  | 4 |  |
| Mean (SD) | 77.3 (15.7) |  | 69.1 (9.8) |  | 75.3 (10.6) |  |
| Median | 75.0 |  | 66.5 |  | 75.5 |  |
| Q1, Q3 | 67.0, 86.0 |  | 62.3, 75.5 |  | 65.3, 85.0 |  |
| Min, max | 53.0, 108.0 |  | 56.0, 88.0 |  | 62.0, 88.0 |  |
| **Week 1^c^** | | | | | | |
| n | 8 | 8 | 11 | 11 | 4 | 4 |
| Mean (SD) | 76.6 (15.8) | −0.3 (1.9) | 68.7 (10.7) | −0.3 (1.6) | 75.3 (11.5) | 0.0 (2.2) |
| Median | 74.0 | 0.0 | 65.0 | −1.0 | 74.5 | 0.5 |
| Q1, Q3 | 64.8, 88.5 | −1.5, 1.0 | 61.0, 81.0 | −1.0, 0.0 | 64.8, 86.5 | −2.3, 1.8 |
| Min, max | 54.0, 104.0 | −4.0, 2.0 | 57.0, 88.0 | −2.0, 4.0 | 62.0, 90.0 | −3.0, 2.0 |
| **Week 2^c^** | | | | | | |
| n | 7 | 7 | 9 | 9 | 4 | 4 |
| Mean (SD) | 76.7 (16.8) | 0.0 (1.9) | 69.0 (10.6) | −1.2 (1.8) | 73.8 (10.3) | −1.5 (1.7) |
| Median | 76.0 | 0.0 | 63.0 | −1.0 | 75.0 | −2.0 |
| Q1, Q3 | 63.0, 84.0 | −2.0, 2.0 | 62.0, 80.0 | −3.0, 0.0 | 63.5, 82.8 | −2.8, 0.3 |
| Min, max | 7 | 7 | 9 | 9 | 4 | 4 |
| **Week 4^c^** | | | | | | |
| n | 6 | 6 | 10 | 10 | 4 | 4 |
| Mean (SD) | 75.8 (17.1) | −1.5 (1.9) | 68.5 (9.5) | −1.7 (2.4) | 74.3 (10.3) | −1.0 (0.8) |
| Median | 77.0 | −1.0 | 65.0 | −1.5 | 75.0 | −1.0 |
| Q1, Q3 | 61.3, 86.5 | −2.8, 0.0 | 62.5, 77.5 | −3.3, 0.3 | 64.3, 83.5 | −1.8, −0.3 |
| Min, max | 53.0, 103.0 | −5.0, 0.0 | 56.0, 84.0 | −6.0, 2.0 | 61.0, 86.0 | −2.0, 0.0 |
| **Week 8^c^** | | | | | | |
| n | 6 | 6 | 10 | 10 | 2 | 2 |
| Mean (SD) | 74.3 (17.3) | −3.0 (2.5) | 67.5 (9.0) | −2.7 (2.8) | 67.5 (6.4) | −1.5 (3.5) |
| Median | 75.0 | −3.5 | 65.5 | −2.0 | 67.5 | −1.5 |
| Q1, Q3 | 58.3, 86.3 | −5.3, 0.0 | 59.5, 76.8 | −5.3, −0.8 | 63.0, 72.0 | −4.0, 1.0 |
| Min, max | 53.0, 102.0 | −6.0, 0.0 | 56.0, 81.0 | −7.0, 2.0 | 63.0, 72.0 | −4.0, 1.0 |
| **EOT Week 12^c^** | | | | | | |
| n | 5 | 5 | 10 | 10 | 3 | 3 |
| Mean (SD) | 73.6 (15.2) | −3.0 (2.7) | 66.4 (8.7) | −3.9 (2.8) | 74.3 (11.0) | −1.0 (2.0) |
| Median | 72.0 | −3.0 | 63.5 | −3.0 | 75.5 | −1.0 |
| Q1, Q3 | 56.5, 91.5 | −5.5, −0.5 | 60.0, 75.8 | −7.0, −1.8 | 63.0, 85.0 | −3.0, 1.0 |
| Min, max | 52.0, 101.0 | −7.0, 0.0 | 57.0, 81.0 | −7.0, 0.0 | 63.0, 85.0 | −3.0, 1.0 |

^a^Laboratory values expressed as “less than” or “greater than” have been imputed using the next numerical value. Patients who received a blood transfusion were censored from their first transfusion date; ^b^Baseline values based on samples taken on Day 1, shortly before administration of first dose; ^c^Values based on samples taken 2 h post dose.

BID, twice daily; ChfB, change from baseline; EOT, end of treatment; MCV, mean corpuscular volume; QD, once daily; Q1, 1^st^ quartile; Q3, 3rd quartile; SD, standard deviation.

## Supplemental Table 7. Summary statistics for LDH (U/L) over time (full analysis set, N=25).^a^

|  | Vamifeport QD (n=9) | | Vamifeport (n=12) | | Placebo (n=4) | |
| --- | --- | --- | --- | --- | --- | --- |
|  | Value | ChfB | Value | ChfB | Value | ChfB |
| **Baseline^b^** | | | | | | |
| n | 9 |  | 12 |  | 4 |  |
| Mean (SD) | 244.3 (89.8) |  | 332.7 (165.0) |  | 230.3 (88.9) |  |
| Median | 252.0 |  | 282.0 |  | 239.0 |  |
| Q1, Q3 | 158.0, 326.5 |  | 197.3, 436.0 |  | 144.8, 307.0 |  |
| Min, max | 142.0, 393.0 |  | 178.0, 666.0 |  | 135.0, 308.0 |  |
| **Week 1^c^** | | | | | | |
| n | 9 | 9 | 12 | 12 | 4 | 4 |
| Mean (SD) | 252.2 (102.1) | 7.9 (17.8) | 326.4 (137.3) | −6.3 (65.7) | 201.5 (67.0) | −28.8 (26.1) |
| Median | 248.0 | 2.0 | 285.0 | −4.5 | 209.0 | −25.5 |
| Q1, Q3 | 166.0, 344.5 | −5.0, 25.0 | 226.5, 416.8 | −55.5, 16.5 | 134.3, 261.3 | −55.3, −5.5 |
| Min, max | 137.0, 432.0 | −9.0, 39.0 | 164.0, 639.0 | −104.0, 158.0 | 122.0, 266.0 | −61.0, −3.0 |
| **Week 2^c^** | | | | | | |
| n | 8 | 8 | 12 | 12 | 4 | 4 |
| Mean (SD) | 224.5 (61.2) | −1.3 (20.4) | 291.5 (119.8) | −41.2 (71.4) | 193.5 (63.6) | −36.8 (35.8) |
| Median | 203.5 | 0.0 | 256.5 | −11.5 | 205.0 | −37.5 |
| Q1, Q3 | 174.5, 289.0 | −21.5, 12.8 | 210.8, 382.8 | −89.3, 4.3 | 127.5, 248.0 | −71.0, −1.8 |
| Min, max | 159.0, 316.0 | −26.0, 31.0 | 152.0, 539.0 | −206.0, 37.0 | 110.0, 254.0 | −78.0, 6.0 |
| **Week 4^c^** | | | | | | |
| n | 6 | 6 | 9 | 9 | 4 | 4 |
| Mean (SD) | 191.8 (34.7) | −0.3 (21.5) | 285.4 (109.0) | −43.8 (62.3) | 215.0 (76.2) | −15.3 (30.8) |
| Median | 177.0 | −0.5 | 283.0 | −13.0 | 221.0 | −10.5 |
| Q1, Q3 | 164.8, 234.0 | −20.3, 19.5 | 198.5, 414.0 | −87.5, −6.5 | 138.5, 285.5 | −46.3, 11.0 |
| Min, max | 161.0, 237.0 | −24.0, 24.0 | 135.0, 424.0 | −174.0, 12.0 | 121.0, 297.0 | −57.0, 17.0 |
| **Week 8^c^** | | | | | | |
| n | 6 | 6 | 10 | 10 | 3 | 3 |
| Mean (SD) | 189.5 (33.1) | −2.7 (21.4) | 284.9 (106.3) | −78.0 (99.6) | 210.3 (63.0) | −38.7 (36.4) |
| Median | 175.0 | −4.5 | 308.0 | −54.5 | 240.0 | −55.0 |
| Q1, Q3 | 164.8, 230.5 | −22.0, 15.3 | 175.8, 390.3 | −130.0, −18.5 | 138.0, 253.0 | −64.0, 3.0 |
| Min, max | 158.0, 232.0 | −22.0, 25.0 | 134.0, 437.0 | −274.0, 36.0 | 138.0, 253.0 | −64, 3 |
| **EOT Week 12^c^** | | | | | | |
| n | 5 | 5 | 11 | 11 | 3 | 3 |
| Mean (SD) | 172.8 (33.0) | −7.4 (15.2) | 282.0 (114.1) | −64.7 (116.5) | 244.0 (94.0) | −5.0 (32.9) |
| Median | 176.0 | −4.0 | 248.0 | −46.0 | 266.0 | 6.0 |
| Q1, Q3 | 143.0, 201.0 | −22.0, 5.5.0 | 184.0, 370.0 | −127.0, 12.0 | 141.0, 325.0 | −42.0, 21.0 |
| Min, max | 142, 223.0 | −30.0, 9,0 | 163.0, 524.0 | −350.0, 81.0 | 141.0, 325.0 | −42.0, 21.0 |

^a^Laboratory values expressed as “less than” or “greater than” have been imputed using the next numerical value. Patients who received a blood transfusion were censored from their first transfusion date; ^b^Baseline values based on samples taken on Day 1, shortly before administration of first dose; ^c^Values based on samples taken 2 h post dose.

BID, twice daily; ChfB, change from baseline; EOT, end of treatment; LDH, lactate dehydrogenase; QD, once daily; Q1, 1^st^ quartile; Q3, 3rd quartile; SD, standard deviation.

## Supplemental Table 8. Summary statistics for unconjugated bilirubin (µmol/L) over time (full analysis set, N=25).^a^

|  | Vamifeport QD (n=9) | | Vamifeport BID (n=12) | | Placebo (n=4) | |
| --- | --- | --- | --- | --- | --- | --- |
|  | Value | ChfB | Value | ChfB | Value | ChfB |
| **Baseline^b^** | | | | | | |
| n | 9 |  | 12 |  | 4 |  |
| Mean (SD) | 29.9 (18.5) |  | 61.9 (33.5) |  | 30.8 (28.2) |  |
| Median | 23.0 |  | 60.5 |  | 21.0 |  |
| Q1, Q3 | 16.5, 45.0 |  | 28.5, 89.5 |  | 11.3, 60.0 |  |
| Min, max | 14.0, 65.0 |  | 20.0, 128.0 |  | 9.0, 72.0 |  |
| **Week 1^c^** | | | | | | |
| n | 9 | 9 | 12 | 12 | 4 | 4 |
| Mean (SD) | 28.9 (19.1) | −1.0 (3.2) | 57.3 (31.9) | −4.7 (10.0) | 32.0 (29.9) | 1.3 (3.3) |
| Median | 24.0 | −1.0 | 60.0 | −4.5 | 22.5 | 1.0 |
| Q1, Q3 | 15.0, 42.5 | −4.5, 1.5 | 22.8, 84.3 | −7.5, 2.8 | 10.0, 63.5 | −1.8, 4.5 |
| Min, max | 11.0, 66.0 | −5.0, 4.0 | 18.0, 107.0 | −25.0, 9.0 | 8.0, 75.0 | −2.0, 5.0 |
| **Week 2^c^** | | | | | | |
| n | 8 | 8 | 10 | 10 | 4 | 4 |
| Mean (SD) | 28.3 (18.8) | −1.1 (2.5) | 44.1 (21.0) | −8.3 (11.4) | 29.8 (28.7) | −1.0 (2.2) |
| Median | 19.0 | −0.5 | 40.5 | −4.0 | 21.5 | −0.5 |
| Q1, Q3 | 15.5, 46.0 | −2.8, 0.8 | 24.0, 68.5 | −21.5, 1.0 | 8.5, 59.3 | −3.3, 0.8 |
| Min, max | 15.0, 63.0 | −6.0, 2.0 | 19.0, 71.0 | −28.0, 4.0 | 5.0, 71.0 | −4.0, 1.0 |
| **Week 4^c^** | | | | | | |
| n | 6 | 6 | 10 | 10 | 4 | 4 |
| Mean (SD) | 25.3 (17.4) | −0.7 (3.3) | 56.7 (32.2) | −11.1 (11.0) | 32.8 (29.2) | 2.0 (2.6) |
| Median | 19.5 | −0.5 | 56.0 | −9.5 | 24.0 | 2.0 |
| Q1, Q3 | 14.8, 33.0 | −3.5, 1.8 | 28.3, 76.0 | −20.8, −0.8 | 11.8, 62.5 | −0.5, 4.5 |
| Min, max | 14.0, 60.0 | −5.0, 4.0 | 14.0, 119.0 | −30.0, 0.0 | 8.0, 75.0 | −1.0, 5.0 |
| **Week 8^c^** | | | | | | |
| n | 6 | 6 | 8 | 8 | 2 | 2 |
| Mean (SD) | 24.0 (14.9) | −2.0 (4.9) | 52.6 (36.1) | −11.6 (14.7) | 37.5 (41.7) | −3.0 (2.8) |
| Median | 18.5 | 0.0 | 50.5 | −7.0 | 37.5 | −3.0 |
| Q1, Q3 | 16.0, 29.3 | −5.8, 1.3 | 23.8, 60.3 | −27.0, 2.0 | 8.0, 67.0 | −5.0, −1.0 |
| Min, max | 16.0, 54.0 | −11.0, 2.0 | 20.0, 132.0 | −33.0, 4.0 | 8.0, 67.0 | −5.0, −1.0 |
| **EOT Week 12^c^** | | | | | | |
| n | 5 | 5 | 10 | 10 | 3 | 3 |
| Mean (SD) | 19.0 (4.2) | 0.8 (3.5) | 61.0 (29.1) | −6.1 (15.6) | 31.3 (29.7) | −1.7 (7.1) |
| Median | 20.0 | 1.0 | 58.5 | −3.0 | 24.0 | −3.0 |
| Q1, Q3 | 15.0, 22.5 | −2.5, 4.0 | 37.8, 94.3 | −12.3, 5.0 | 6.0, 64.0 | −8.0, 6.0 |
| Min, max | 15.0, 25.0 | −4.0, 5.0 | 21.0, 102.0 | −34.0, 11.0 | 6.0, 64.0 | −8.0, 6.0 |

^a^Laboratory values expressed as “less than” or “greater than” have been imputed using the next numerical value. Patients who received a blood transfusion were censored from their first transfusion date; ^b^Baseline values based on samples taken on Day 1, shortly before administration of first dose; ^c^Values based on samples taken 2 h post dose.

BID, twice daily; ChfB, change from baseline; EOT, end of treatment; QD, once daily; Q1, 1^st^ quartile; Q3, 3rd quartile; SD, standard deviation.

## Supplemental Figure 1. Box plots of mean (SD) change from baseline in hepcidin levels, over the 12-week treatment period (full analysis set, N=25).


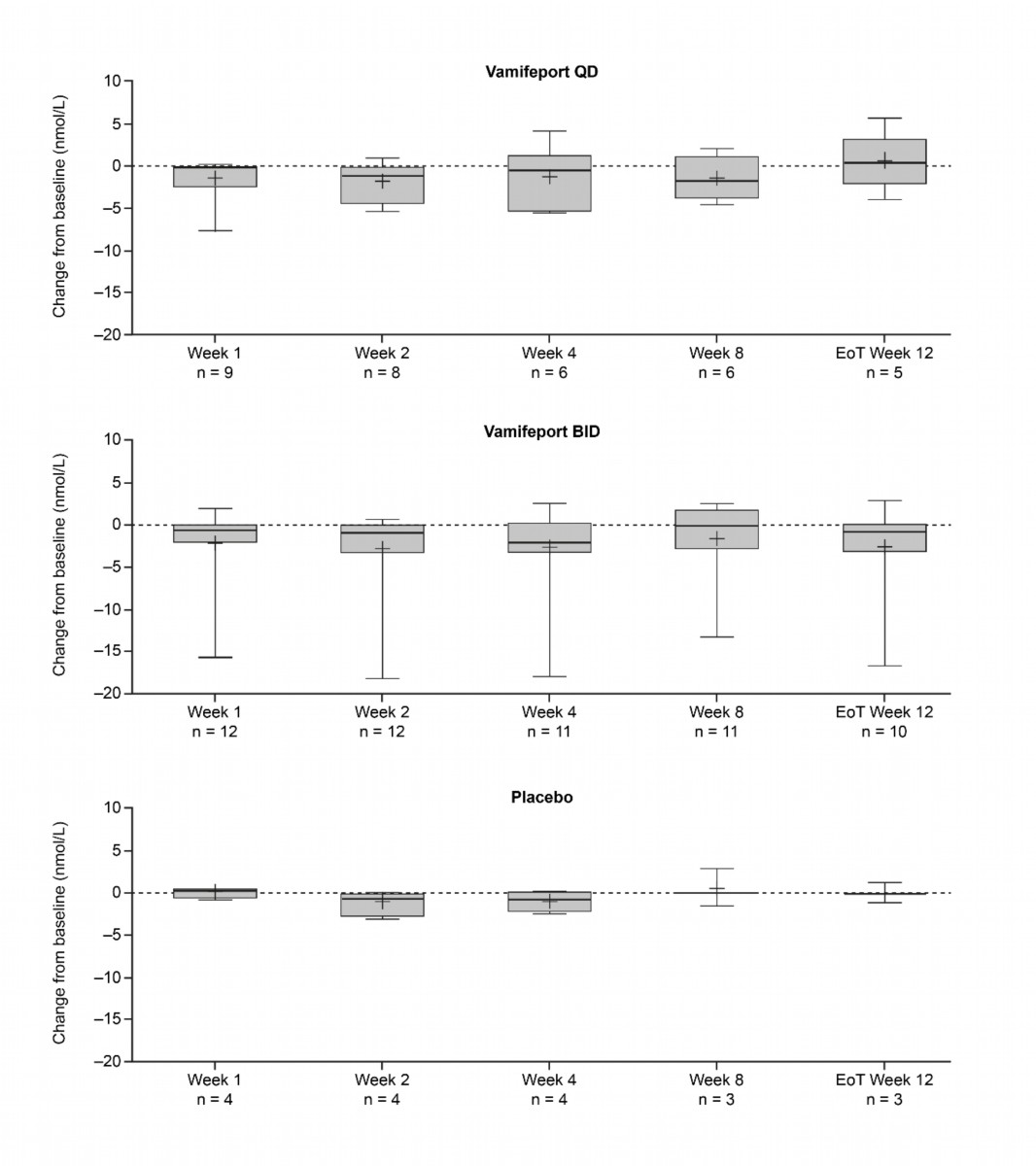


BID, twice daily; EoT, end of treatment; QD, once daily; SD, standard deviation.

## Supplemental Figure 2. Individual patient plots of TSAT% over time by treatment (full analysis set, N=25).

Notes: BID=Twice daily; QD=Once daily; TSAT=Transferrin saturation.

## Supplemental Figure 3. Individual patient plots of relative fetal hemoglobin expression over time by treatment (full analysis set, N=25).


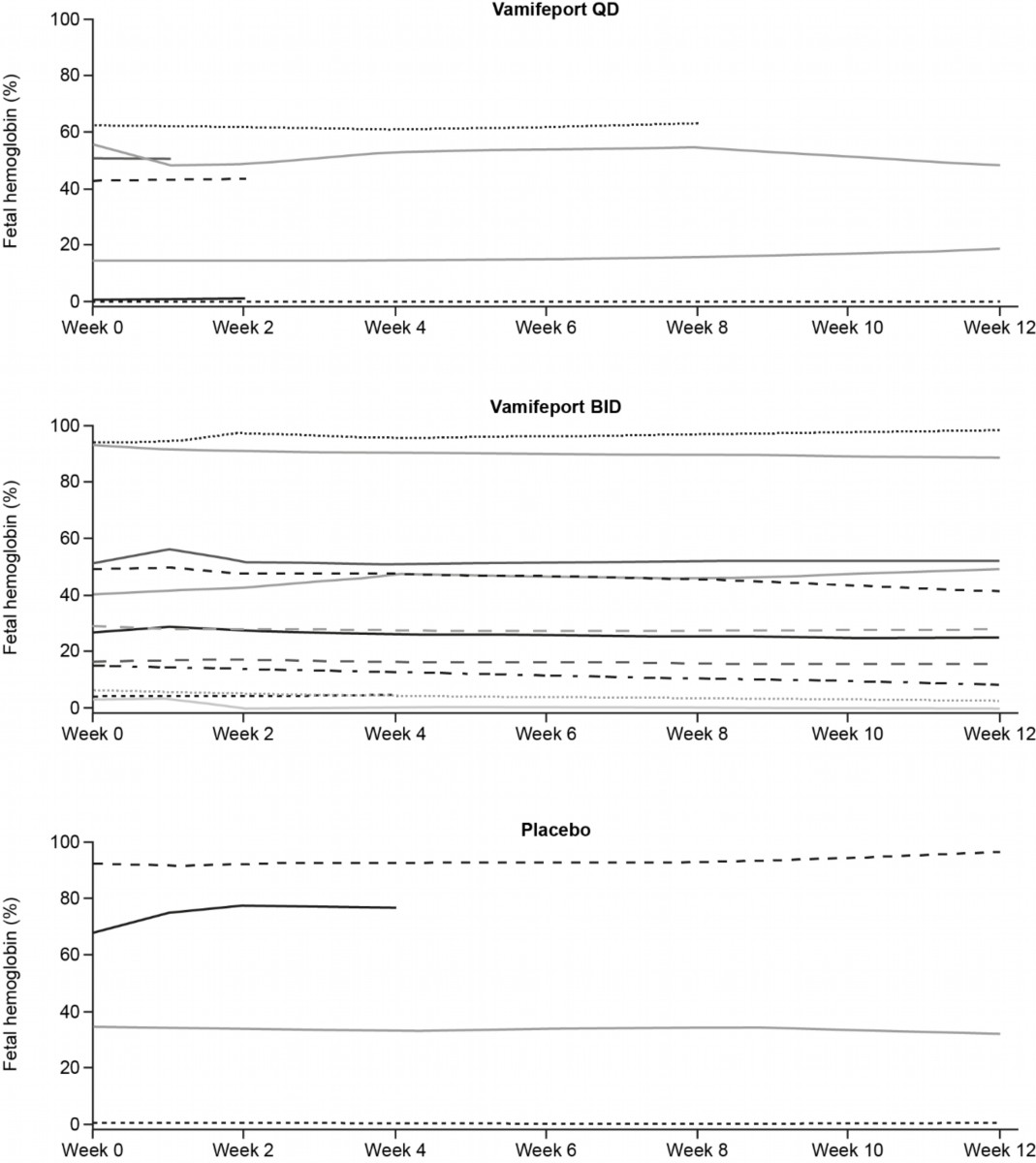


BID, twice daily; QD, once daily.

## Supplemental Figure 4. Box plots of RBC counts (mean and SD), over the 12-week treatment period (full analysis set, N=25).

## Supplemental Figure 5. Box plots of mean (SD) change from baseline in RBC counts, over the 12-week treatment period (full analysis set, N=25).

## Supplemental Figure 6. Box plots of reticulocyte counts (mean and SD), over the 12-week treatment period (full analysis set, N=25).

## Supplemental Figure 7. Box plots of mean (SD) change from baseline in reticulocyte counts, over the 12-week treatment period (full analysis set, N=25).
